# Neutrophil-to-lymphocyte ratio as a predictive biomarker for hyperprogressive disease mediated by immune checkpoint inhibitors: a systematic review and meta-analysis

**DOI:** 10.3389/fimmu.2024.1393925

**Published:** 2024-09-23

**Authors:** Bo Pei, Jue Zhang, Lin Lai, Hui Chen

**Affiliations:** ^1^ Department of Oncology, The Central Hospital of Enshi Tujia and Miao Autonomous Prefecture, Enshi Clinical College of Wuhan University, Enshi, China; ^2^ Department of Radiation Oncology and Medical Oncology, Zhongnan Hospital of Wuhan University, Wuhan, China; ^3^ Department of Radiology, The First People’s Hospital of Tianmen City, Tianmen, China; ^4^ Department of Immunology, Wuhan University TaiKang Medical School (School of Basic Medical Sciences), Wuhan, China

**Keywords:** hyperprogressive disease, immunotherapy, immune checkpoint inhibitors, meta-analysis, neutrophil-to-lymphocyte ratio

## Abstract

**Background:**

Hyperprogressive disease (HPD) is a novel pattern of paradoxically rapid tumor progression, which often leads to early death, mostly in the first 2 months of treatment with immune checkpoint inhibitors (ICIs). Currently, there is no validated biomarker to assess patients at risk of HPD.

**Aim:**

The aim of this study was to systematically evaluate the predictive value of the neutrophil-to-lymphocyte ratio (NLR) in HPD and establish a reliable variable to support clinicians in defining personalized treatment strategies.

**Methods:**

PubMed, Embase, Web of Science, Scopus, and Cochrane Library databases were searched for studies published before 31 December 2023. The Newcastle–Ottawa Scale (NOS) was used to evaluate the quality of eligible studies. The pooled odds ratios (ORs) and 95% confidence intervals (CIs) were calculated using a random-effects or a fixed-effects model to evaluate the association between the NLR and the risk of HPD.

**Results:**

A total of 17 studies with 2,964 patients were included for meta-analysis. The incidence of HPD across different types of tumors ranged from 6.3% to 35.6%. In the pooled analysis of the NLR and HPD, we identified that the NLR significantly associated with the risk of HPD (OR = 0.65; 95% CI: 0.46 to 0.91; *p* = 0.01) (*I* ^2^ = 52%, *p* = 0.007).

**Conclusion:**

In the future, the NLR may serve as a remarkable biomarker for predicting the risk of HPD in clinical practice.

## 1 Introduction

Cancer ranks as one of the most fatal diseases, with its incidence rapidly growing, and remains a major public health problem in the world (1, 2). Although a variety of treatment methods have been applied to cancer, including surgery, chemotherapy, radiotherapy, and targeted therapies over the past few decades, the prognosis remains unsatisfactory (2). Fortunately, the advent of immunotherapy has revolutionized cancer treatment and has been the cornerstone of success in treating several malignancies (3, 4). Immune checkpoint inhibitors (ICIs) have become a novel and effective therapeutic strategy and the mainstay of treatment for many cancers (5, 6). Despite proven clinical efficacy, a significant proportion of patients do not respond to immunotherapy. A subset of patients who experienced an extremely rapid boost in tumor volume during the treatment with ICIs that far exceeded the pretreatment growth rate, which was defined as hyperprogressive disease (7). Patients with HPD often showed both shorter progression-free survival and overall survival than patients with natural progressive disease (PD) and loss of eligibility for subsequent systemic treatments owing to clinical deterioration (8). Thus, early identification of HPD is very crucial. Multiple studies have reported possible predictive factors of HPD, including serum lactate dehydrogenase above the upper normal limit, the presence of more than two metastatic sites, liver metastases, programmed cell death ligand-1 (PD-L1) positivity, a Royal Marsden Hospital prognostic score of 2 or above, and an Eastern Cooperative Oncology Group Performance Score ≥ 2 (9, 10). Apart from these factors, numerous studies have been conducted to evaluate the neutrophil-to-lymphocyte ratio (NLR) for HPD (11–28). However, these studies have produced different results, and many do not support each other. The predictive value of the NLR for HPD remains controversial. Thus, we performed a systematic review and meta-analysis on this topic to identify the predictive value of HPD.

## 2 Materials and methods

### 2.1 Literature search strategy and eligibility criteria

This systematic review and meta-analysis was conducted based on PRISMA guidelines (29). Two investigators (BP and HC) independently searched PubMed, Embase, Web of Science, Scopus, and Cochrane Library databases for studies published before 31 December 2023. The following keywords were used for the search: immunotherapy, programmed death receptor-1, PD-1, programmed cell death-ligand 1, PD-L1, cytotoxic T-lymphocyte-associated protein 4, CTLA-4, ipilimumab, tremelimumab, nivolumab, pembrolizumab, ICI, hyperprogression, and HPD. Additional studies were selected for full-text review by exploring the references and relevant reviews cited in the selected articles. Articles that were published in English with full texts. Finally, we reviewed the list of retrieved articles to select potentially relevant literature and discussed differences in specific studies, which were then resolved with the consensus of both investigators.

The inclusion criteria were as follows: (1) prospective or retrospective studies that reported the characteristics of patients who developed HPD during immunotherapy, regardless of tumor type; (2) all patients were diagnosed as having malignant tumors by biopsy; and (3) the value of the NLR was calculated according to the level of neutrophils and lymphocytes.

The exclusion criteria included the following: (1) duplicate studies, reviews, case reports, letters, reference abstracts, or full text unavailable in English; (2) non-human studies, such as *in vitro* or animal studies; or (3) studies that did not provide the value of the NLR.

### 2.2 Data extraction and quality assessment

From each study, BP and HC extracted the name of the study, first author and year of publication, study design, country and institution, underlying malignancy, treatment regimen (PD-1/PD-L1 inhibitor monotherapy or combined with other therapies), drugs, the definition of HPD, total number of HPD cases and groups, and NLR cutoff values. When duplicate publications were identified, we included only the most recent and complete reports of controlled trials.

Quality assessments were assessed using the Newcastle–Ottawa Scale (NOS) (30), which evaluated the study design based on eight aspects: population selection, comparability, and exposure. Studies with final scores of 6 to 9 were regarded as high quality.

### 2.3 Statistical analysis

The pooled odds ratios (ORs) and 95% confidence intervals (CIs) were calculated using a random-effects or a fixed-effects model to evaluate the association between the NLR and the risk of HPD. We performed the *χ* ^2^-based *Q* test to assess interstudy heterogeneity and calculated the *I*² statistic, representing the percentage of total variability observed due to study heterogeneity. The heterogeneity between studies was considered to indicate a statistically significant difference with heterogeneity *p* < 0.1 or *I* ^2^ > 50%. Publication biases were evaluated with funnel plots. Furthermore, Egger’s and Begg’s tests would proceed if required. In addition, each study was individually excluded from the meta-analysis for sensitivity analyses (31). All statistical analyses were performed with Revman ver.5.3.

## 3 Results

### 3.1 The characteristics of the included studies

**Figure 1:**
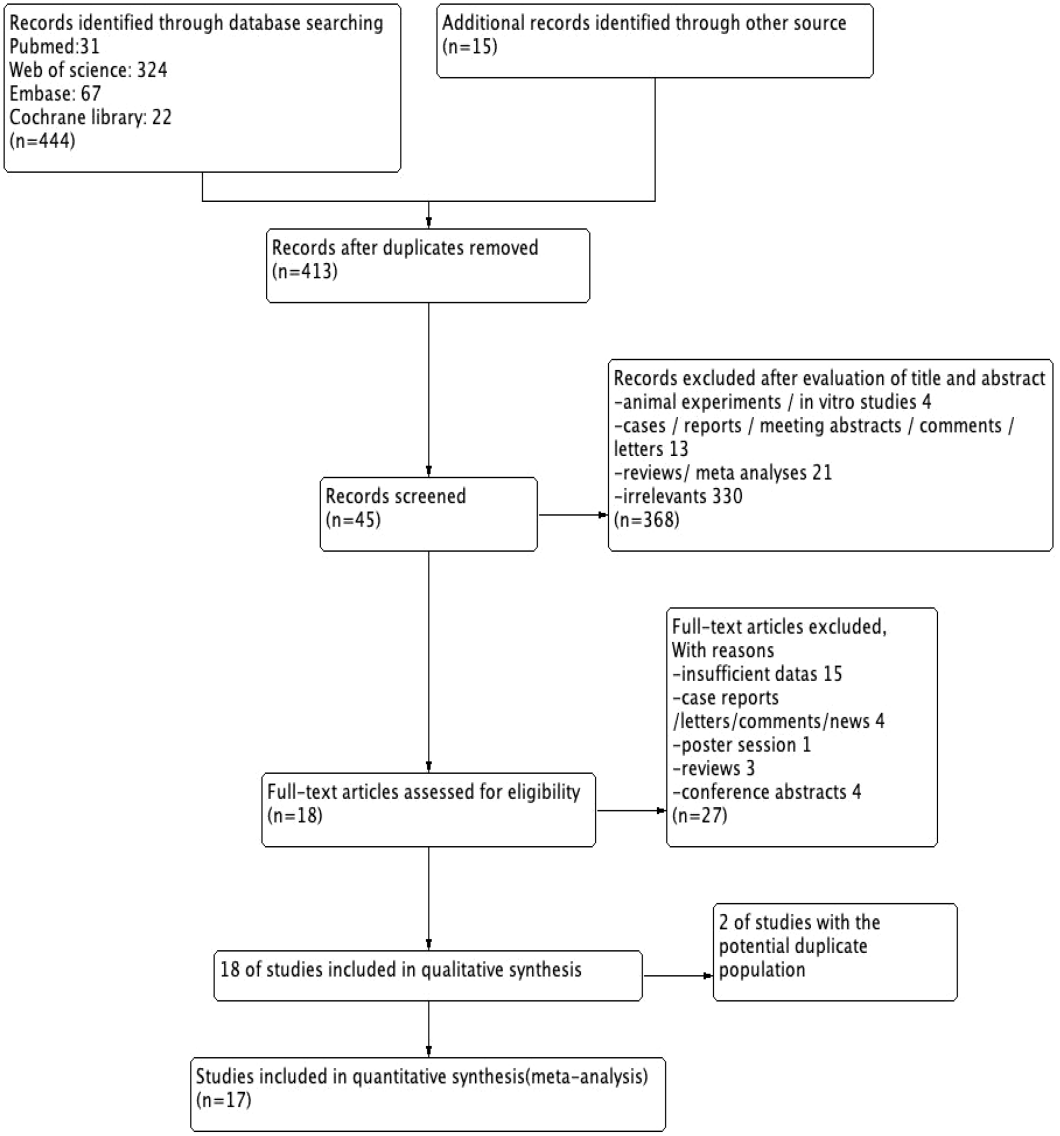
Flowchart of study selection.

A total of 459 articles were reviewed, and 45 potentially relevant articles were screened after full-text screening. Finally, 18 studies (3,370 patients) fulfilled the inclusion criteria for abstracts and full article reviews (11–28). From the 18 studies, we found that 2 potentially eligible studies (15, 20) were performed in the same group, and only the most recent (20) was included to avoid duplication. Therefore, a total of 17 studies were included for this meta-analysis, with publication dates ranging from 2018 to 2023 (**Figure 1**).

The sample size enrolled in each trial ranged between 51 and 406. We found 10 studies with PD-1/PD-L1 inhibitor monotherapy, including nivolumab, atezolizumab, pembrolizumab, durvalumab, avelumab, and camrelizumab, while the other 7 studies have PD-1/PD-L1 inhibitor monotherapy or in combination with CTLA-4 or other ICIs. Among all patients included, 1,210 were diagnosed with non-small cell lung cancer (NSCLC), 245 were diagnosed with recurrent and/or metastatic head and neck squamous carcinoma (R/M HNSCC), 283 were diagnosed with liver cancer (LC), and the rest were diagnosed with other cancers (**Table 1**). The incidence of HPD across different types of tumors ranged from 6.3% to 35.6% in cohorts of patients with NSCLC, 16.7%–27.5% for AGC, 14.4%–18.3% for R/M HNSCC, 10% for breast cancer, and 9.4%–14.5% for liver cancer. Additionally, we found that all identified eligible studies were retrospective.

**Table 1:** Characteristics of the enrolled studies: population characteristics.

Study	Year	Study design	Country, institution	Sample size	Underlying malignancy	Treatment	Incidence of HPD
Yildirim, H.C.	2022	Retrospective cohort	Turkey, single center	121	RCC (33%); melanoma (34%); NSCLC (17%); others (16%)	PD-1/PD-L1 inhibitor monotherapy	20/121 (16.5%)
Zhang, L.	2021	Retrospective cohort	China, single center	69	HCC	PD-1 inhibitors monotherapy (nivolumab, pembrolizumab, and camrelizumab)	10/69 (14.5%)
Xiao, L.S.	2021	Retrospective cohort	China, single center	129	PLC	PD-1/PD-L1 inhibitor monotherapy	13/129 (10.1%)
Petrova, M.P.	2020	Retrospective cohort	Bulgaria, 5 centers	167	NSCLC	Pembrolizumab	16/167 (9.6%)
Kim, C.G.	2019	Retrospective cohort	Korea, single center	263	NSCLC	PD-1/PD-L1 inhibitor monotherapy	54/263 (20.5%)
Kim, Y.	2019	Retrospective cohort	Korea, single center	135	NSCLC	PD-1/PD-L1 inhibitor monotherapy (nivolumab, pembrolizumab, atezolizumab, durvalumab, and avelumab)	48/135 (35.6%)
Chen, S.	2021	Retrospective cohort	China, single center	377	LC (35%); PC (7%); GC (9%); others (56%);	PD-1 inhibitors monotherapy or combination (nivolumab/pembrolizumab)	38/377 (10.1%)
Karabajakian, A.	2020	Retrospective analysis of clinical trials	France, single center	120	R/M HNSCC	PD1/PD-L1 inhibitors monotherapy or in combination with CTLA4 or KIR antibody	22/120 (18.3%)
Ferrara, R.	2020	Retrospective cohort	France, 8 centers	406	NSCLC	PD-1/PD-L1 inhibitor monotherapy or combination (nivolumab, pembrolizumab, atezolizumab, and durvalumab)	56/406 (13.8%)
Kim, J.	2022	Retrospective cohort	Korea, 8 centers	219	NSCLC	PD-1/PD-L1 inhibitor monotherapy (nivolumab, pembrolizumab, atezolizumab, and durvalumab)	35/219 (15.9%)
Wang, Z.	2021	Retrospective cohort	China, single center	51	GC	PD-1/PD-L1 inhibitors monotherapy	14/51 (27.5%)
Park, J.H.	2020	Retrospective cohort	Korea, 11 centers	125	R/M HNSCC	PD-1/PD-L1/CTLA4 inhibitors monotherapy or combination	18/125 (14.4%)
Masabiko, A.	2023	Retrospective cohort	Japanese, 24 centers	245	GC	PD-1: nivolumab	41/245 (16.7%)
Sae, Y.	2023	Retrospective cohort	Japanese 6 centers	85	HCC	PD-L1+VGFR: Atez/Bev	8/85 (9.4%)
Yildirim, H.C.	2022	Retrospective cohort	Turkey, single center	95	Any cancer subtype	PD-1/PD-L1 inhibitor monotherapy or combination	26/95 (27.4%)
Igracio, M.	2020	Retrospective cohort	Spain, single center	221	Different tumor types	PD-1/PD-L1 inhibitors monotherapy or combination	14/221 (6.3%)
Takaomi, H.	2020	Retrospective cohort	Japanese, 23 centers	136	GC	PD-1: nivolumab	30/136 (22.1%)

HPD, hyperprogressive disease; NSCLC, non-small cell lung cancer; LC, lung cancer; RCC, renal cell carcinoma; HCC, hepatocellular carcinoma; PLC, primary liver cancer; GC, gastrointestinal adenocarcinoma; R/M HNSCC, recurrent and/or metastatic head and neck squamous carcinoma; PD-1, programmed cell death protein 1; PD-L1, programmed death 1 ligand 1; CTLA4, cytotoxic T-lymphocyte antigen 4; KIR, killer immunoglobulin like receptor.

The definition of HPD varied across the included studies due to the lack of standard criteria. The criteria reported by the studies included in this analysis were adopted (**Table 2**). Among these studies, Yildirim et al. and Petrova et al. adopted criteria that combined clinical and radiologic parameters (11, 14). Other studies evaluated the acceleration of tumor growth with volume or the sum of the largest diameters based on three imaging time points (pretreatment, baseline, and posttreatment). It should be noted that the definition of tumor growth kinetics (TGK) was also different in the studies of Kim CG (16) and Kim Y (17). Kim CG and colleagues defined TGK as the difference in the sum of the longest diameter of the target lesions according to RECIST 1.1 each month, whereas it was defined by Kim Y and colleagues as the difference in the total tumor volume of the target lesions per unit time. Moreover, the cutoff values of the NLR were different for the enrolled studies, and the value of 3 was most commonly used.

**Table 2:** Characteristics of the enrolled studies: definition and cutoff.

Study	Year	Definition of HPD	Cutoff value for NLR
Yildirim, H.C.	2022	Fulfilling at least three of the following five criteria: (1) TTF < 2 months; (2) >50% increase in the sum of target lesion major diameters between baseline and first radiologic evaluation; (3) appearance of at least two new lesions in an organ already involved between baseline and first radiologic evaluation; (4) spread of the disease to a new organ between baseline and first radiologic evaluation; and (5) ECOG ≥2 during the first 2 months of treatment.	5
Zhang, L.	2021	Defined as follows: (1) time to treatment failure (TTF)^a^ < 2 months; (2) disease progression at the first evaluation and >50% increase in TGRa; and (3) TGRpost/TGRpreb ≥2.	3.57
Xiao, L.S.	2021	Defined HPD as PD within approximately 2 months after the initiation of treatment according to the RECIST 1.1, with a measurable lesion increase of ≥10 mm. The criteria for HPD were as follows: (1) the total diameter of the target lesion increased by ≥40% compared with baseline and/or (2) the total diameter of the target lesion increased by ≥20% compared with baseline and new lesions appeared in at least two different organs.	3
Petrova, M.P.	2020	Fulfilling at least three of the following five criteria: (1) time to treatment failure < 3 months; (2) increase ≥ 50% in the sum of target lesion major diameters between baseline and first radiological evaluation; (3) appearance of at least two new lesions in an organ already involved between baseline and first radiological evaluation; (4) spread of the disease to a new organ between baseline and first radiological evaluation; and (5) clinical deterioration with ECOG ≥ 2 during the first 3 months of treatment. Criteria 1 and 5 were mandatory.	5
Kim, C.G.	2019	Defined as TGRpost/TGRpre^b^ ≥2 and TGKpost/TGKpre^c^ ≥ 2 according to the RECIST 1.1 and TTF <2 months.	3
Kim, Y.	2019	Defined as follows: (1) TTF <2 months; (2) TGKpost/TGKpre^c^ ≥ 2; and (3) volume increase of 50% compared with baseline.	4
Chen, S.	2021	TGRpost−TGRpre^b^ > 50%	3
Karabajakian, A.	2020	TGKpost/TGKpre^c^ ≥ 2	NA
Ferrara, R.	2020	Defined as RECIST version 1.1 progression at first CT scan with TGRpost−TGRpre^b^ > 50%	3
Kim, J.	2022	TGKpost/TGKpre^c^ ≥ 2 and TTF <2 months	3.3
Wang, Z.	2021	TGKpost/TGKprec > 2	3.14
Park, J.H.	2020	TGKpost/TGKprec > 2	4
Masabiko, A.	2023	Defined as a ≥2-fold increase in the tumor growth rate of measurable lesions	1.8
Sae, Y.	2023	TGR ≥ 2 or TGK ≥ 2	3.43
Yildirim, H.C.	2022	Defined by RECIST progression and at least three of the following symptoms: time to treatment failure <2 months (time to treatment failure is defined as the time from the start of treatment with ICI to ICI discontinuation for any reason); increase of ≥50% in the sum of target lesion major diameters between baseline and first radiologic evaluation; the appearance of at least two new lesions in an organ already involved between baseline and first radiologic evaluation; spread of the disease to a new organ between baseline and first radiologic evaluation; and clinical deterioration with a decrease in ECOG performance status ≥2 during the first 2 months of treatment	3.375
Igracio, M.	2020	Defined HPD based on RECIST as PD in the first 8 weeks after treatment initiation and minimum increase in the measurable lesions of 10 mm plus (i) increase of ≥40% in sum of target lesions compared with baseline [which represents doubling in unidimensional target lesions compared with classic RECIST PD criterion (20%)]; and/or (ii) increase of ≥20% in sum of target lesions compared with baseline (the classic RECIST PD criterion) plus the appearance of new lesions in at least two different organs.	3
Takaomi, H.	2020	TGK ratio ≥2 and (SPOST/S0-1) > 0.5	2.4

^a^ TGR was calculated only with measurable target lesions, and based on the sum of the longest diameter or the volume of the target lesion described in the RECIST 1.1 version; ^b^ TGRpost/TGRpre stands for the ratio of TGR after the initiation of experimental treatment to TGR before the initiation of experimental treatment. TGRpost−TGRpre > 50% stands for an absolute increase in the TGR exceeding 50% per month. ^c^ Tumor growth kinetics (TGK) was defined as the change in the sum of the longest diameters of the target lesions according to RECIST 1.1 criteria per month. TGKpost/TGKpre stands for the ratio of TGK after the initiation of experimental treatment to TGK before the initiation of experimental treatment. Abbreviations: HPD, hyperprogressive disease; ECOG, Eastern Cooperative Oncology Group; NA, not available; TGK, tumor growth kinetics; TGR, tumor growth rate; S0 and SPOST represented the sum of the longest diameters of the target lesions according to RECISTv1.1 and at pre-baseline, baseline, and post CT, respectively. TTF, time to treatment failure.

### 3.2 Relationship between the NLR and HPD in cancers treated with immunotherapy

**Figure 2:**
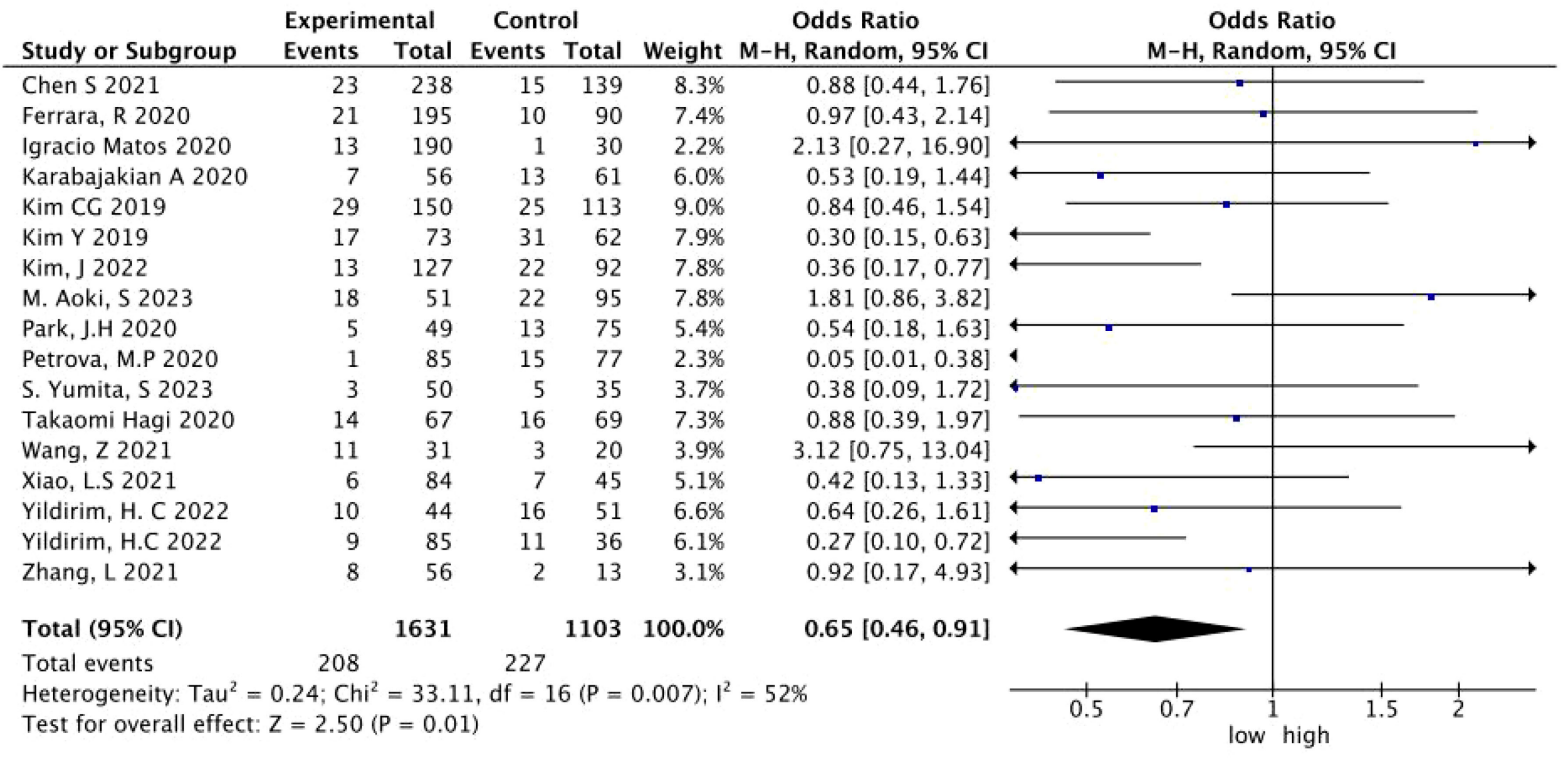
A forest plot of the association between the NLR and HPD in patients treated with immunotherapy. NLR, neutrophil-to-lymphocyte ratio; HPD, hyperprogressive disease; CI, confidence interval.

**Figure 3:**
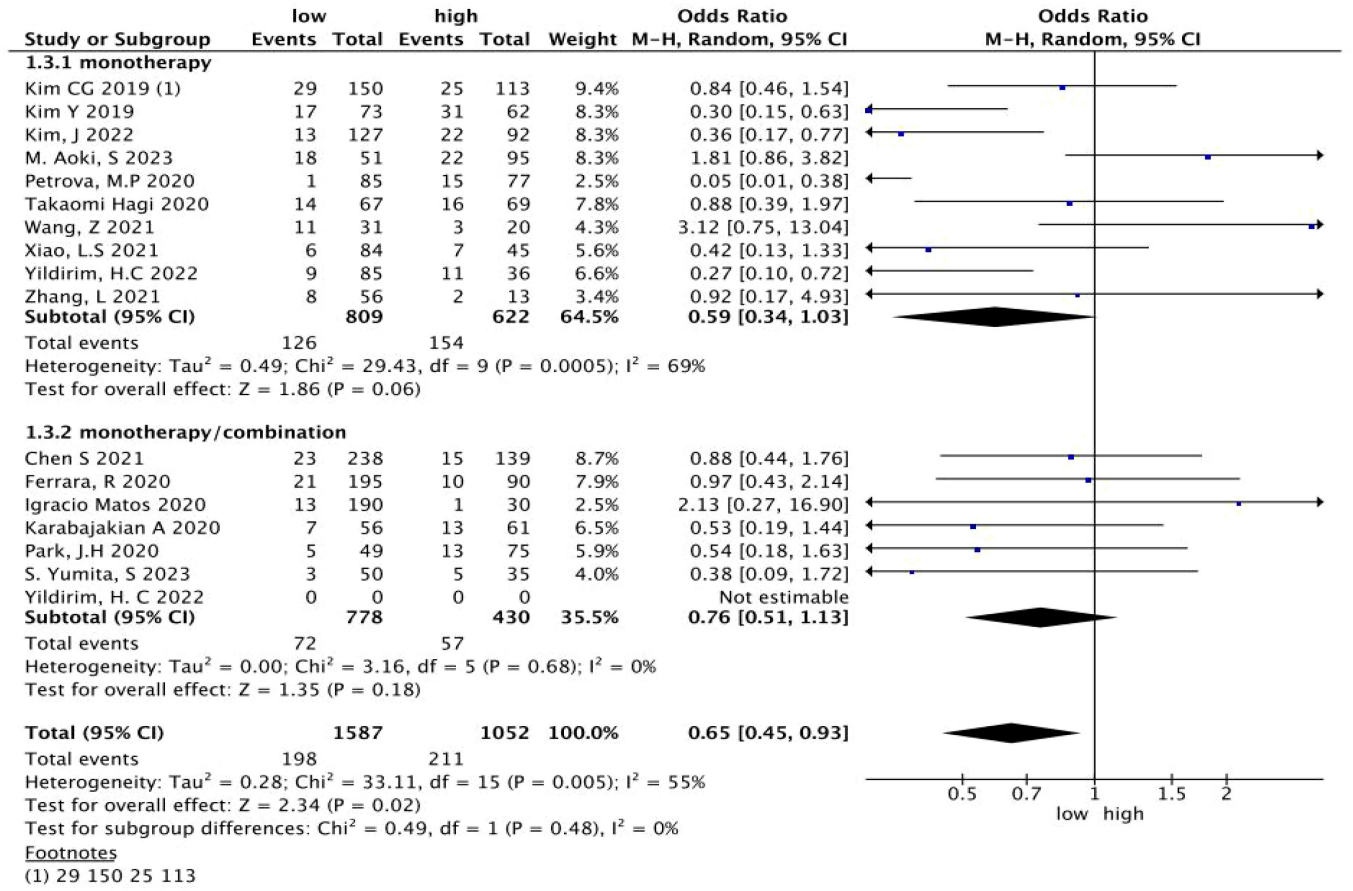
A subgroup analysis of the relationship between the NLR and HPD in patients treated with immunotherapy. NLR, neutrophil-to-lymphocyte ratio; HPD, hyperprogressive disease; CI, confidence interval.

A total of 17 studies with 2,964 patients treated with immunotherapy provided the NLR values and the number of HPD. The data of ORs and 95% CIs from these studies were combined. In the pooled analysis of the NLR and HPD, we identified that the NLR significantly associated with the risk of HPD (OR = 0.65; 95% CI: 0.46 to 0.91; *p* = 0.01) (*I* ^2^ = 52%, *p* = 0.007) (**Figure 2**). To detect the source of heterogeneity, we performed subset analyses on certain clinical factors that might influence the final result, such as the treatment regimen (**Figure 3**).

### 3.3 Sensitivity analysis and publication bias

We found moderate heterogeneity among studies (*p* = 0.007; *I* ^2^ = 52%). Therefore, we performed a sensitivity analysis on all included studies. Each study was individually excluded from the meta-analysis to evaluate the effect of each study on the pooled OR value. The sensitivity analysis results showed that the combined ORs in our meta-analysis were robust (**Supplementary Figures 1**-**17**). As can be seen from **Figure 4**, all included studies were symmetrically distributed on the left and right sides of the inverted funnel plot, suggesting that no potential publication bias was identified.

**Figure 4:**
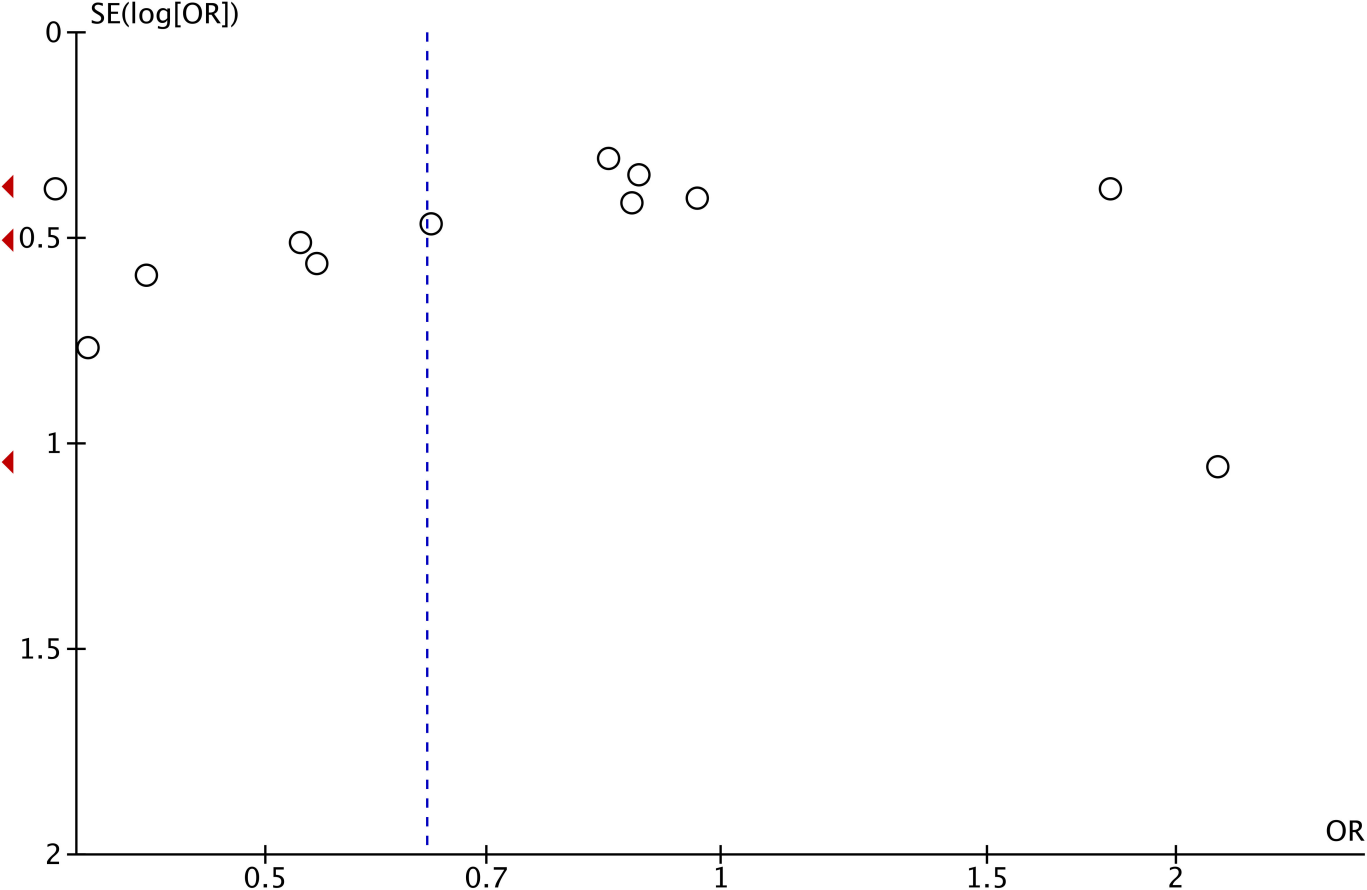
A funnel plot of meta-analysis of the association of the NLR with HPD. NLR, neutrophil-to-lymphocyte ratio; HPD, hyperprogressive disease.

## 4 Discussion

HPD is a paradoxical acceleration of tumor growth phenomenon, which often leads to early death commonly in the first 2 months of treatment with ICIs (32). The incidence of HPD after treatment with ICIs ranged from 1.2% to 43.1% (10), while in our meta-analysis, the pooled incidence of HPD varied from 6.3% to 35.6%, which may be related to the inclusion of different tumor types and different assessment methods for HPD in previous studies (33). Multiple factors are thought to contribute to the occurrence of HPD, including clinical features, genetic features, and tumor immune microenvironment characteristics (9, 10, 34, 35). The mechanism of HPD occurrence and the exact causes are poorly understood. In some preclinical models, ICI causes HPD. Recently, Li et al. found a triple-high gene signature score (IFNγ–FGF2–β-catenin) associated with HPD in patients and revealed crosstalk between metabolic, immunogenic, and oncogenic pathways underlying HPD associated with immunotherapy (36).

HPD is not unique to immunotherapy (37), the incidence of which will increase. It also occurs in conventional chemotherapy and targeted therapies. The overall survival of patients with HPD was significantly shortened, suggesting that once HPD occurs, it can seriously affect the prognosis of patients (38, 39). Although some researchers caution that HPD represents the natural disease course of a subset of patients with aggressive cancers (40–42), most studies now support that patients with HPD had a worse outcome than patients with so-called “conventional” progression (43). Once these patients develop HPD after receiving immunotherapy, they are often deprived of their chance to receive subsequent treatment (44). It is a particular phenomenon that patients cannot benefit from immunotherapy because of the lack of prediction methods (45). A recent study identifies SAA1 and specific metabolomic signatures as potential predictive biomarkers for HPD in patients undergoing immunotherapy across various cancers (46). However, metabolic biomarkers are preferred for the dynamic monitoring of HPD due to their susceptibility to fluctuations caused by metabolic disorders; they will take a long time and may delay HPD diagnosis. Furthermore, many immunohistochemistry specimens cannot be obtained due to various reasons. In this scenario, it is important to identify HPD biomarkers for selection before ICI therapy.

The NLR is defined as the absolute neutrophil count divided by the absolute lymphocyte count. It is a practical, easy acquisition, and low-cost marker of the immune system’s inflammatory response (47). In the oncological context, an elevated NLR is often observed in individuals presenting with advanced or aggressive forms of cancer (48). This may be due to tumor cells secreting granulocyte colony-stimulating factor (G-CSF) and/or granulocyte monocyte colony-stimulating factor (GM-CSF), which not only are direct growth factors for tumor cells, but also may lead to increased NLR in patients. Thus, bone marrow hematopoiesis shifts from the lymphocyte lineage to the granulocyte lineage (49, 50). Accumulating evidence has revealed that the NLR is associated with tumor malignant degree invasive biological features and the efficacy of immunotherapy (51). In the era of immunotherapy, the NLR often represents a significant, feasible prognostic factor (52, 53). An evidence synthesis from 30 meta-analyses indicated that the NLR is associated with poor outcomes in patients with cancer receiving immunotherapy (54). There may be a certain correlation, and a similar situation exists in HPD. An increasing number of researchers have speculated that the NLR may be a biomarker for predicting the occurrence of HPD. However, inconsistent results have emerged from multiple studies. For example, original cohort studies conducted by Petrova et al. (14), Karabajakian et al. (19), Kim et al. (21), Takahashi et al. (55), Milla et al. (56), and Maesaka et al. (57) have concluded that the risk of HPD occurrence will increase with elevated NLR, which can be used as a marker for HPD. Our study was in line with this conclusion. We found that patients with high NLR had a higher probability of developing HPD after ICI treatment, and the difference was statistically significant (OR = 0.65; 95% CI: 0.46 to 0.891; *p* = 0.01). However, the other subset of original cohort studies had been conducted by Champiat et al. (7), Kim et al. (8), Zhang et al. (12), Xiao et al. (13), Chen et al. (18), Ferrara et al. (20), Wang et al. (22), Park et al. (23), and Choi et al. (58), and their colleagues showed that elevated NLR was not significantly associated with the occurrence of HPD. Meanwhile, two meta-analyses had drawn similar conclusions (10, 59). Numerous factors may account for these results. First, some studies included a small sample size. Second, different researchers included different cancer types; for instance, Liu et al. only included solid tumors, and we added hematologic tumors to our study. Last but not least, there is currently no consensus cutoff value of the NLR for predicting the occurrence of HPD. Future studies with larger sample sizes and an optimal cutoff value of the NLR to validate the results of this study are needed.

The exact mechanism between an elevated NLR and HPD has yet to be elucidated. The possible mechanisms are as follows: first, tumor cells can induce bone marrow to produce more neutrophils into the blood and promote tumor progression through multiple immunosuppressive pathways. Neutrophils can interact with other immune cells, such as macrophages and myeloid-derived suppressor cells (MDSCs), to create an immunosuppressive environment. These interactions can lead to the polarization of macrophages towards an M2 phenotype, which is immunosuppressive and supports tumor progression (60). Second, a high NLR often indicates a greater presence of neutrophils, which induce angiogenesis, tumor growth, and metastasis by secreting tumor growth factors, cytokines, and chemokines, such as transforming growth factor P (TGP-P), vascular endothelial growth factor (VEGF), interleukin-6 (IL-6), interleukin-8 (IL-8), interleukin-12 (IL-12), and stromal metalloproteinases (61–63). Third, neutrophils are part of the innate immune system and can contribute to an inflammatory environment that supports tumor progression. An elevated NLR may signal a pro-inflammatory state within the tumor (64). Additionally, neutrophils have been shown to participate in immune evasion strategies employed by tumors. They can release factors that inhibit T-cell function, such as arginase and reactive oxygen species (ROS), which can suppress the activity of cytotoxic T cells that are essential for antitumor immunity (65, 66). Last but not the least, in the latest study, Ng et al. revealed that tumor-reprogrammed neutrophils converge immature and mature neutrophils to a terminally differentiated T3 state, which promotes angiogenesis by localizing in hypoxic–glycolytic niches and enhancing blood vessel formation in the tumor microenvironment, thus promoting the occurrence and development of tumors (67). At the same time, lymphocytes play a vital role in antitumor immunity and are the leading performers of immune functions in the antitumor process. T lymphocytes can recognize and kill tumor cells, thus inhibiting tumor proliferation. Lymphocytes also participate in antitumor immunity by releasing cytokines (68). Since ICIs depend on the suppressive signaling function of T lymphocytes, a decrease in lymphocyte counts decreases the antitumor immune response and affects the efficacy of ICIs. Increased lymphocyte infiltration in the tumor immune microenvironment is associated with better prognosis and immunotherapy efficacy (69, 70).While the number of lymphocytes is insufficient, it leads to an inadequate immune response to the tumor, which promotes tumor progression and metastasis. Increased neutrophils suggest a poor prognosis, while tumor-associated lymphocytes are associated with a better prognosis. The NLR can measure the immune system’s inflammatory state, reflecting the balance between tumor protection and destruction. Therefore, it is inferred that the NLR can be used as a predictor for the occurrence of HPD.

Admittedly, there are several limitations in our study. The meta-analysis included 17 studies, all of which were retrospective studies, and the type of studies in this category was somewhat confounded with bias. It is insufficient to reveal a causal relationship between specific indicators or clinical features and HPD, yet such high-quality prospective studies examining the interaction of these indicators with HPD events are still lacking. At the same time, the assessment criteria for HPD, tumor growth kinetic indicators, and the definition of HPD vary, which may affect the consolidation of results across studies and lead to selection and reporting bias. Thus, we should develop and validate a standardized HPD definition as early as possible in further studies and conduct more high-quality, large-scale multi-center studies to confirm the predictive value of the NLR for the occurrence of HPD and provide strong evidence for clinical decision-making.

Given the limitations of ICI due to HPD incidence, there is an urgent need for reliable biomarkers to predict the occurrence of HPD and the efficacy of ICI (71). Although radiomic features can help identify the features of HPD after immunotherapy has been reported (72), the definition of HPD based on TGR ratio or TKR ratio may preclude its clinical use in many patients due to the lack of pre-baseline imaging data. Such complexity may be the barrier in incorporating the HPD concept into clinical practice. It is imperative for us to search a simpler and more routinely available tool to assess HPD. To a certain extent, the NLR can compensate for the deficiency of imaging examinations, achieve dynamic monitoring, and identify HPD early and accurately, which may reduce the incidence of HPD. For patients who have developed HPD, the combination with other ICIs, radiotherapy, chemotherapy, and targeted therapies may provide a synergistic antitumor effect and become a reliable approach to treating HPD. The study of Park and colleagues suggested that patients who experienced HPD with ICIs should not be excluded from the subsequent salvage chemotherapy treatments, owing to potentially enriched therapeutic benefits of post-ICI chemotherapy in R/M-HNSCC (23). Clinical studies of multiple combination treatment options are currently being explored, and the results of these studies are expected to bring new hope for the clinical treatment of HPD.

In summary, this systematic review and meta-analysis revealed that the NLR might be an easy, low-cost, and readily available method and biomarker to predict the occurrence of HPD. It can be used to identify HPD early and take measures to reduce the incidence of HPD. The result is of great importance for evidence-based clinical decision-making in oncology practice, where immunotherapy has become a mainstay of cancer medical therapy. Therefore, before patients receive ICI therapy, combining routine clinical examinations with an evaluation of NLR is needed to provide them with a safe and optimal treatment option.

## Data Availability

The original contributions presented in the study are included in the article/**Supplementary Material**. Further inquiries can be directed to the corresponding author.
