# Supplementary figures and images for: Neutrophil-to-lymphocyte ratio as a predictive biomarker for hyperprogressive disease mediated by immune checkpoint inhibitors: a systematic review and meta-analysis
Source: Front Immunol. 2024 Sep 23;15:1393925. doi: 10.3389/fimmu.2024.1393925 (PMC11460549; doi:10.3389/fimmu.2024.1393925)

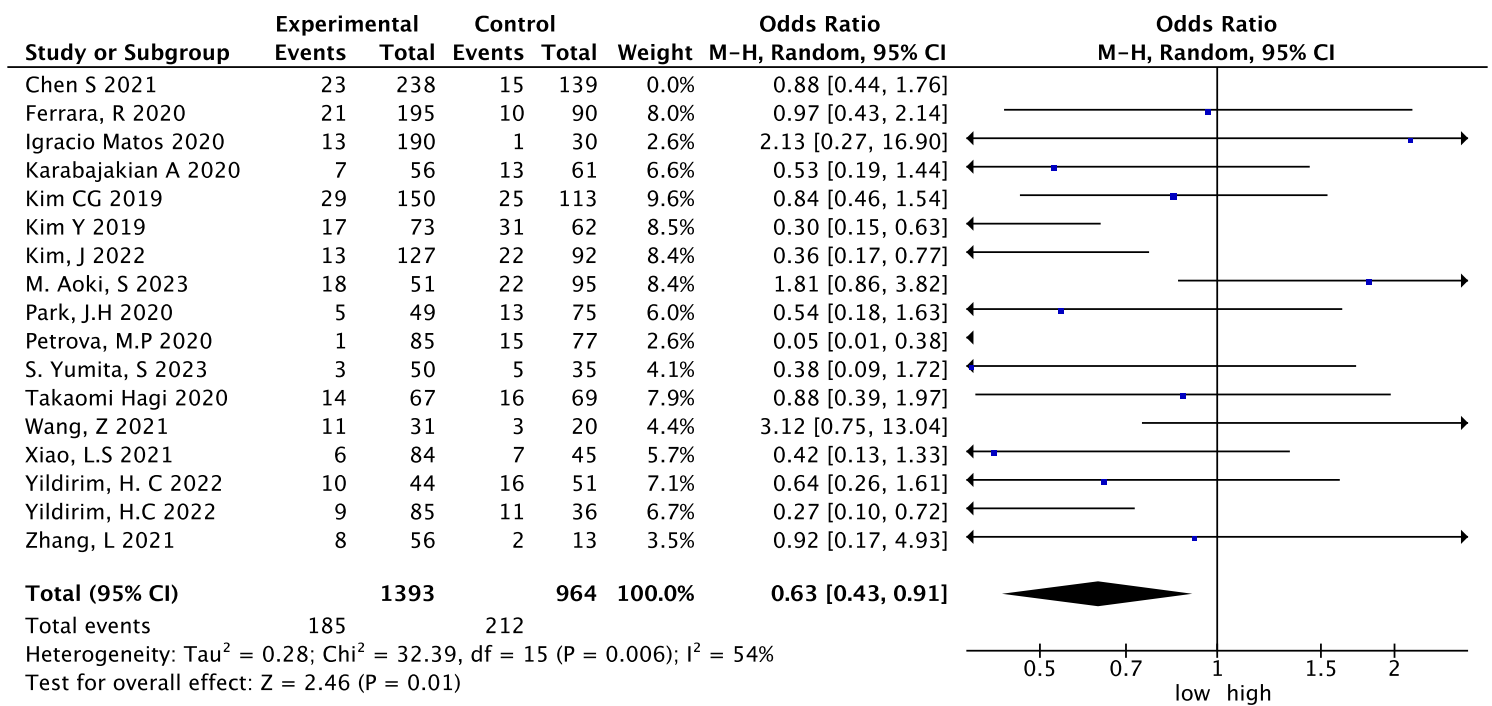

Supplement: Supplementary file 1 [file DataSheet1.zip › Supplementary Figure 1-17/Supplementary Figure 1.pdf]

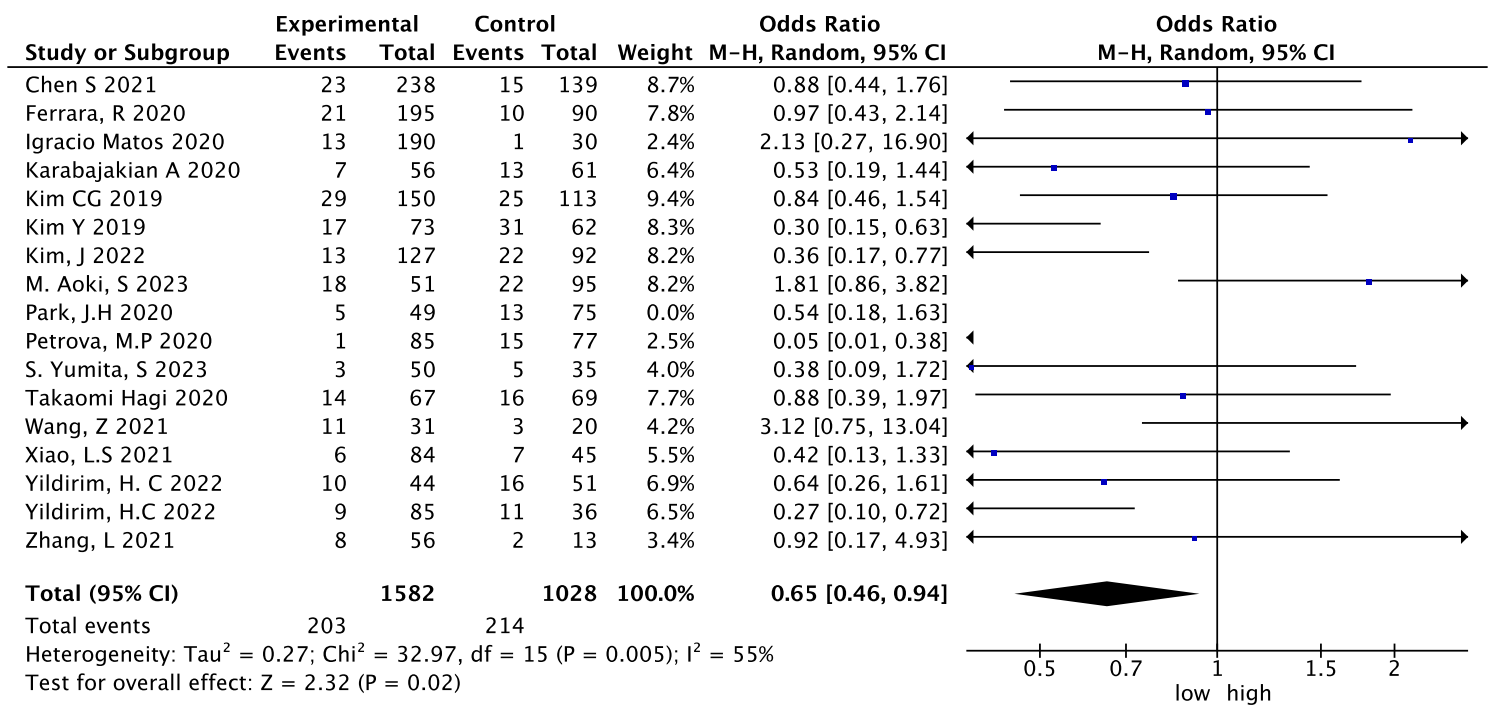

Supplement: Supplementary file 1 [file DataSheet1.zip › Supplementary Figure 1-17/Supplementary Figure 10.pdf]

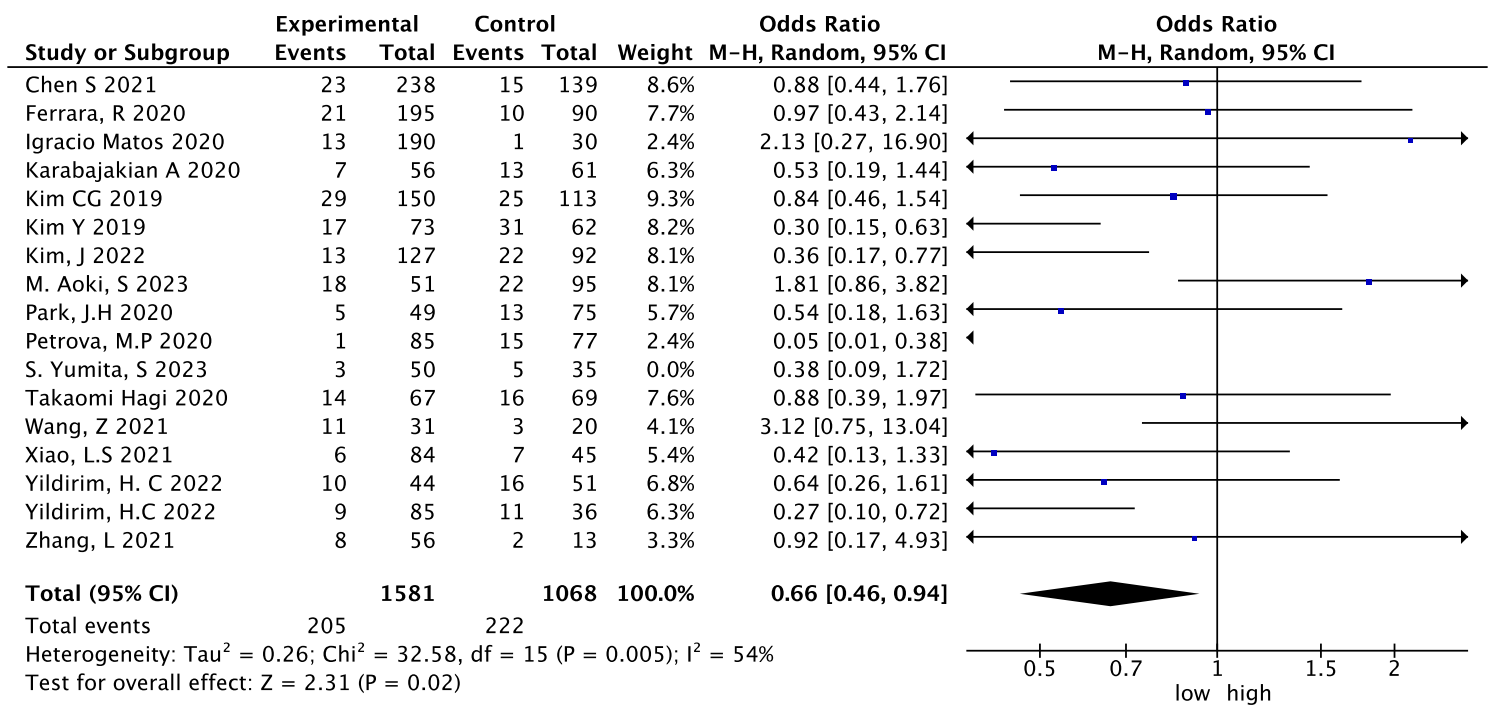

Supplement: Supplementary file 1 [file DataSheet1.zip › Supplementary Figure 1-17/Supplementary Figure 11.pdf]

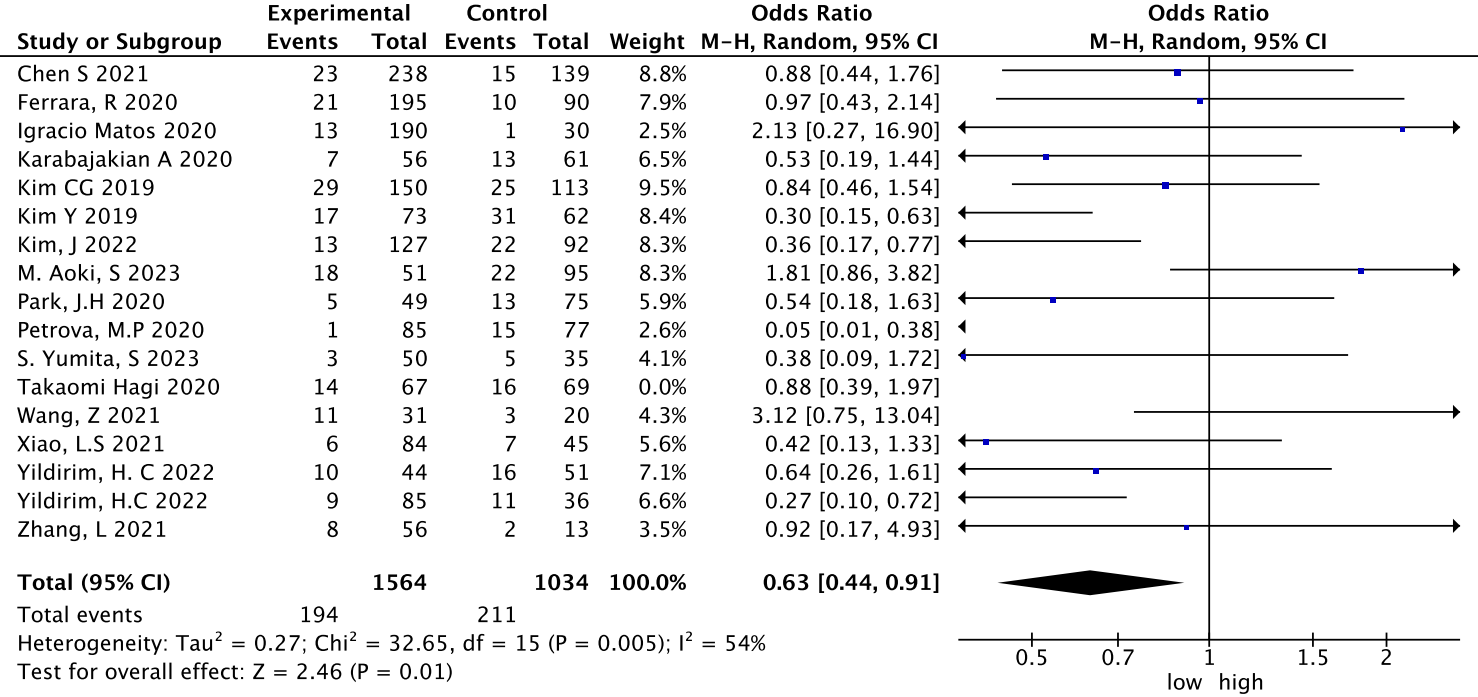

Supplement: Supplementary file 1 [file DataSheet1.zip › Supplementary Figure 1-17/Supplementary Figure 12.pdf]

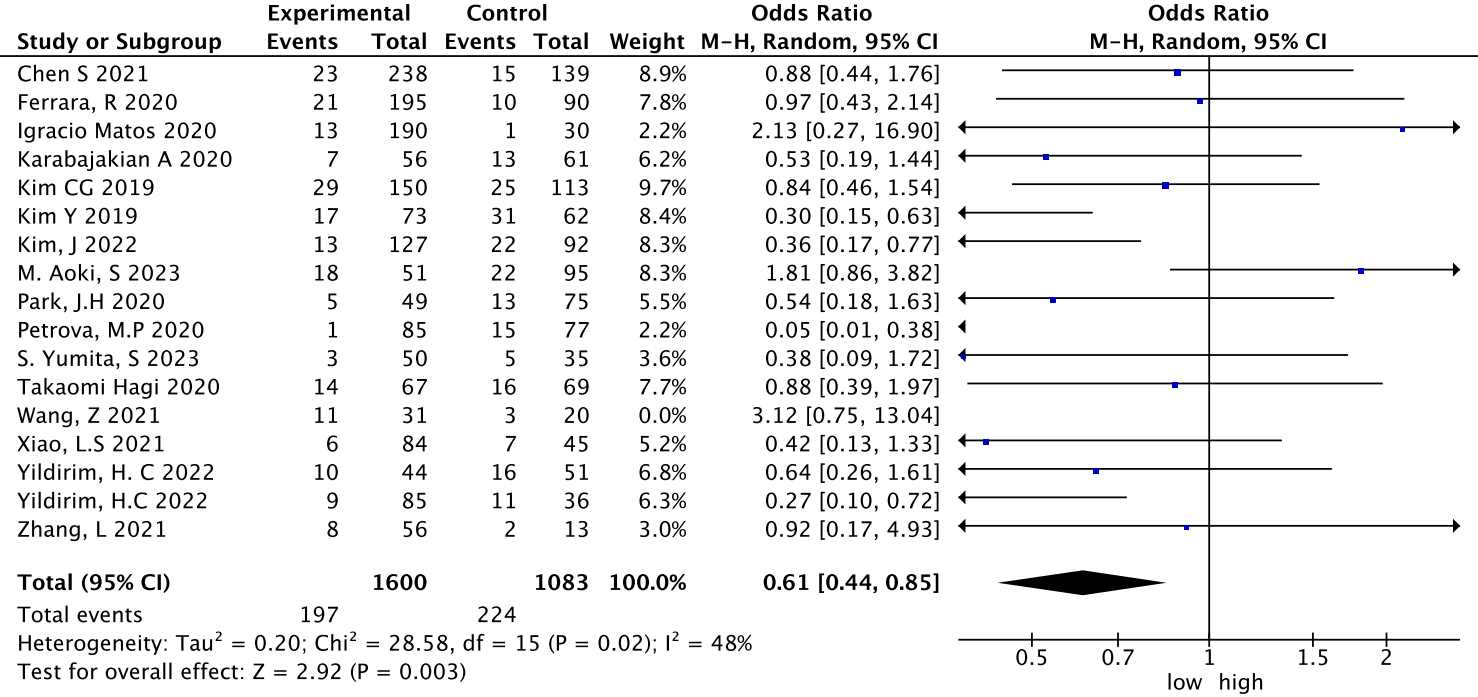

Supplement: Supplementary file 1 [file DataSheet1.zip › Supplementary Figure 1-17/Supplementary Figure 13.pdf]

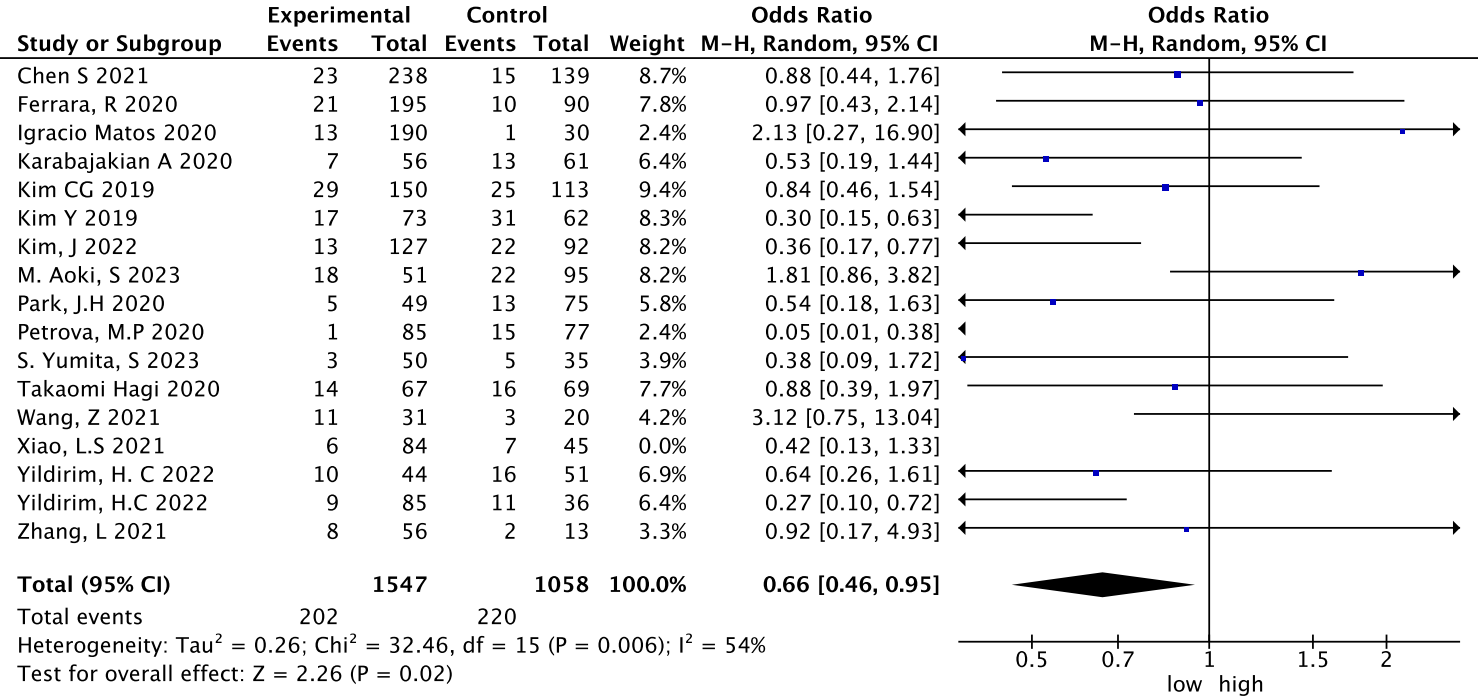

Supplement: Supplementary file 1 [file DataSheet1.zip › Supplementary Figure 1-17/Supplementary Figure 14.pdf]

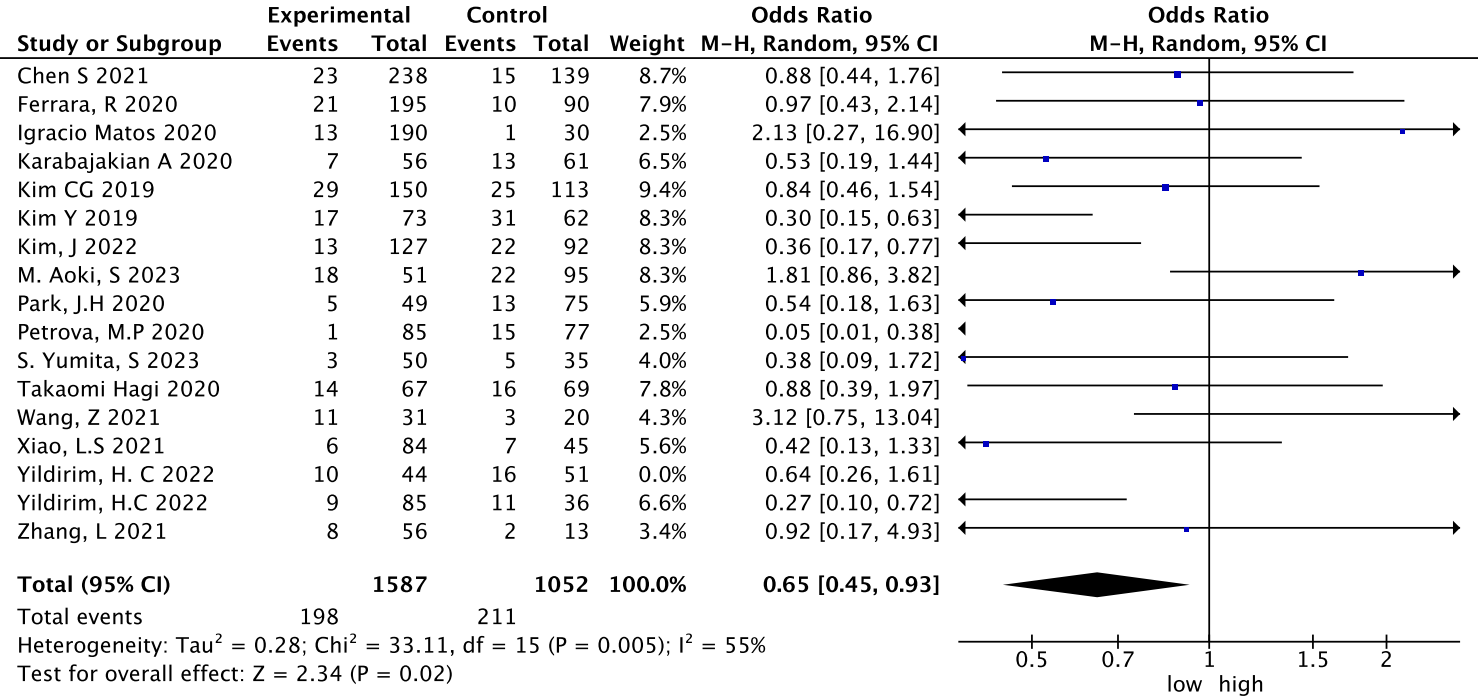

Supplement: Supplementary file 1 [file DataSheet1.zip › Supplementary Figure 1-17/Supplementary Figure 15.pdf]

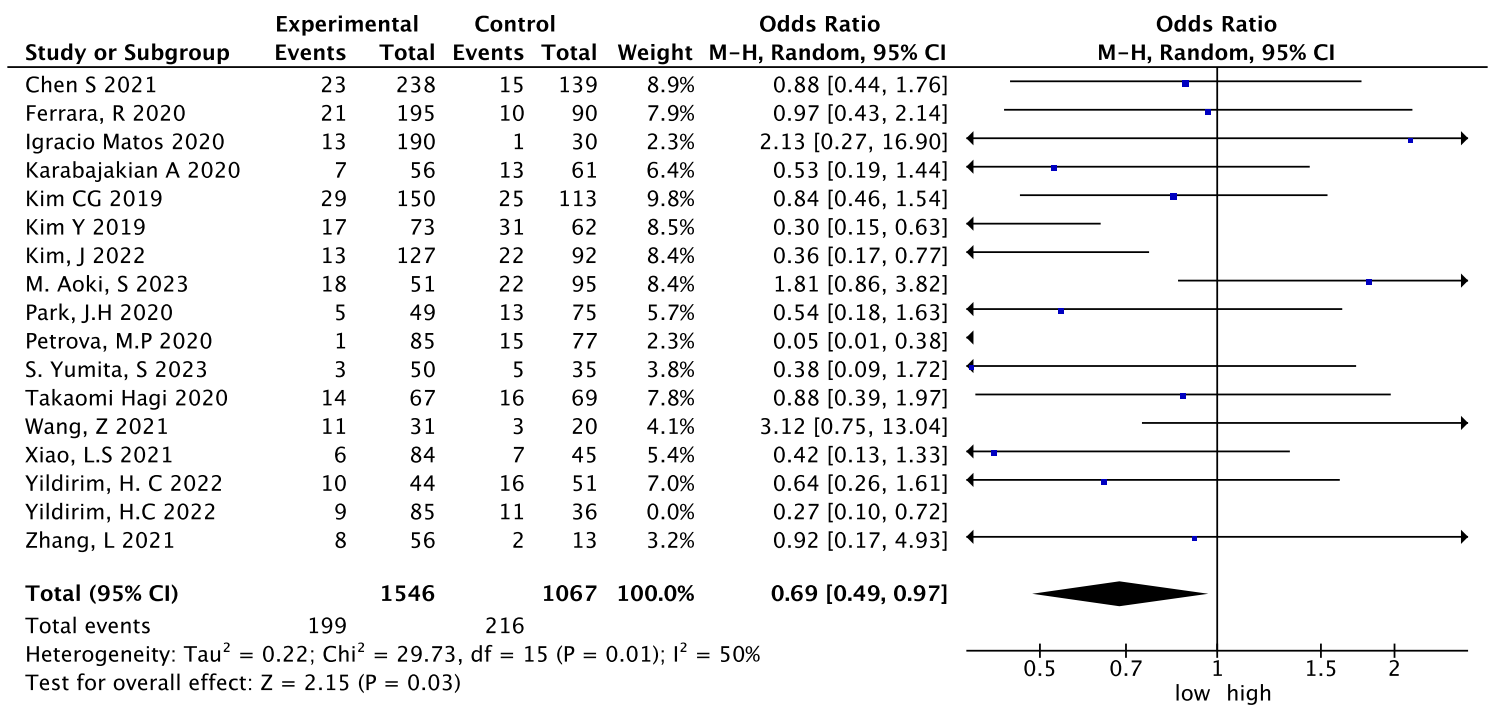

Supplement: Supplementary file 1 [file DataSheet1.zip › Supplementary Figure 1-17/Supplementary Figure 16.pdf]

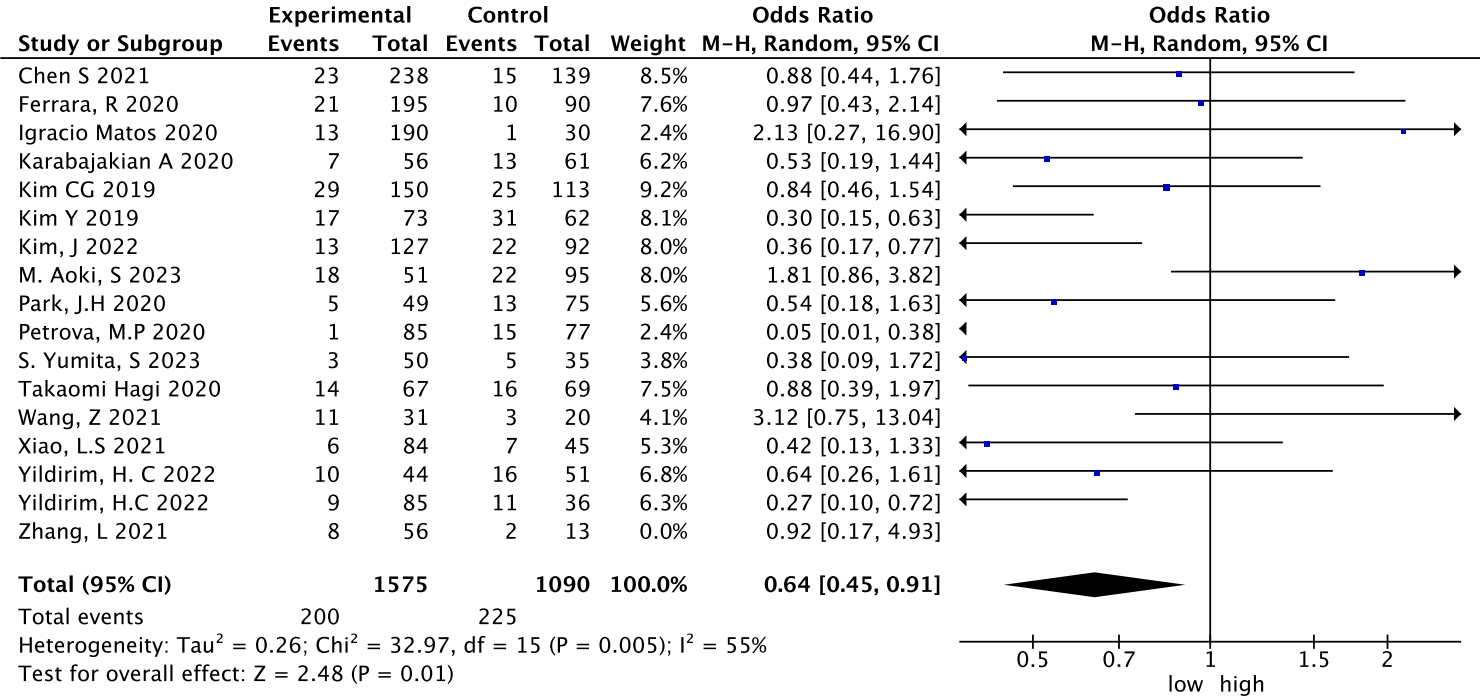

Supplement: Supplementary file 1 [file DataSheet1.zip › Supplementary Figure 1-17/Supplementary Figure 17.pdf]

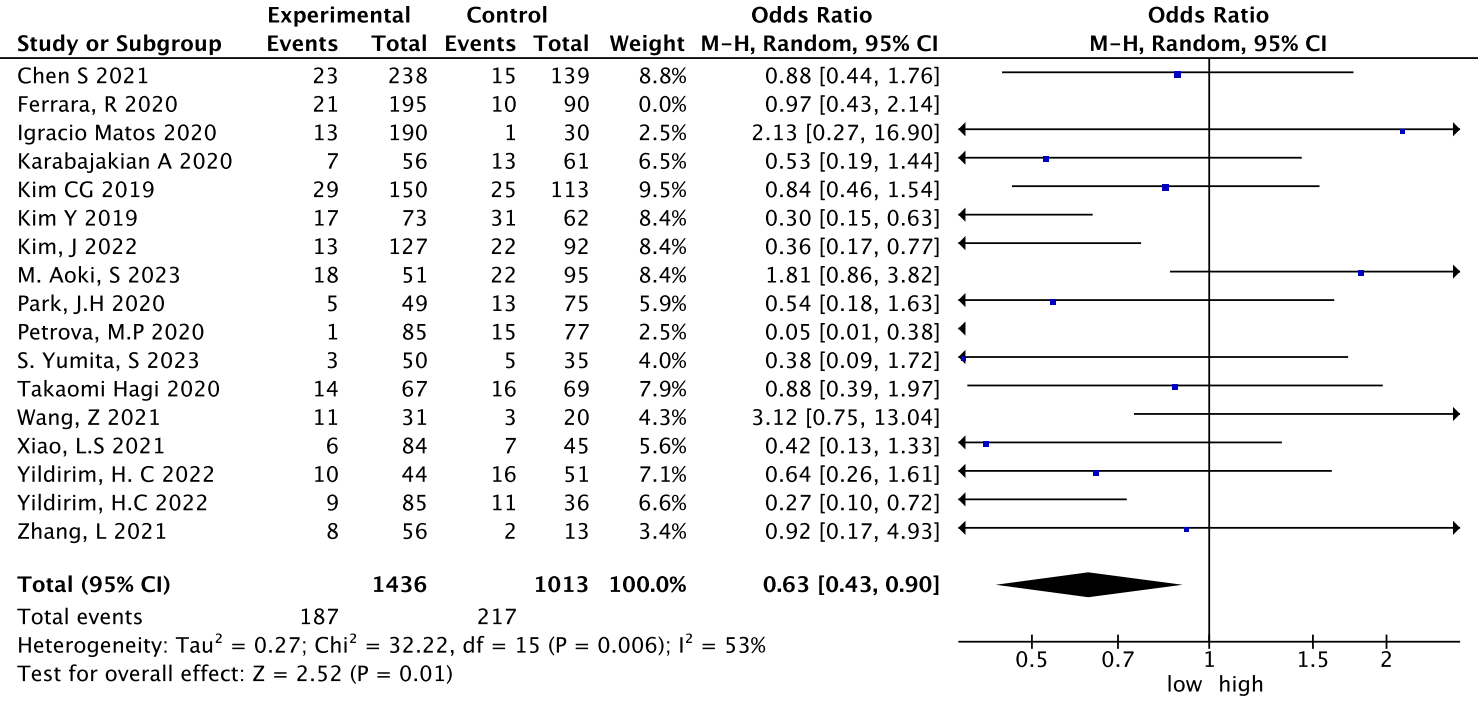

Supplement: Supplementary file 1 [file DataSheet1.zip › Supplementary Figure 1-17/Supplementary Figure 2.pdf]

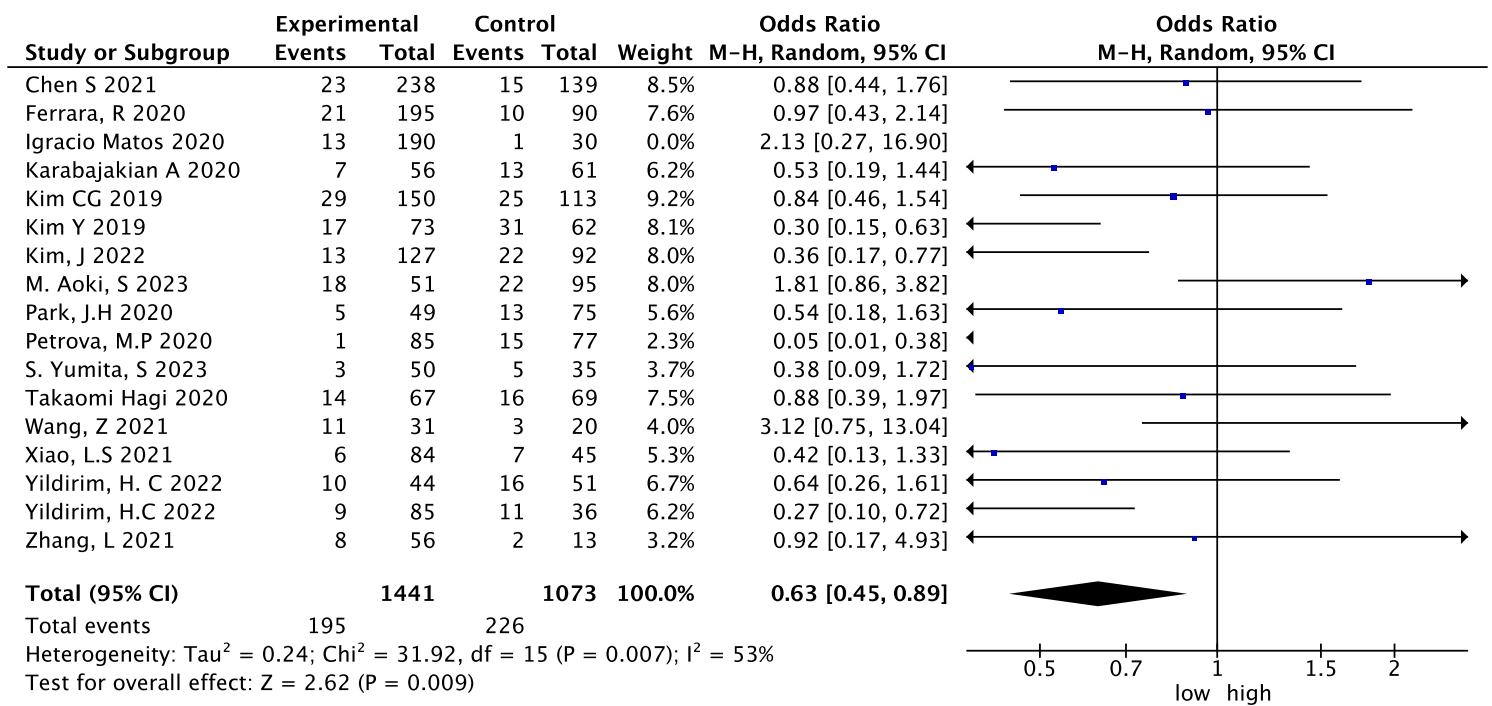

Supplement: Supplementary file 1 [file DataSheet1.zip › Supplementary Figure 1-17/Supplementary Figure 3.pdf]

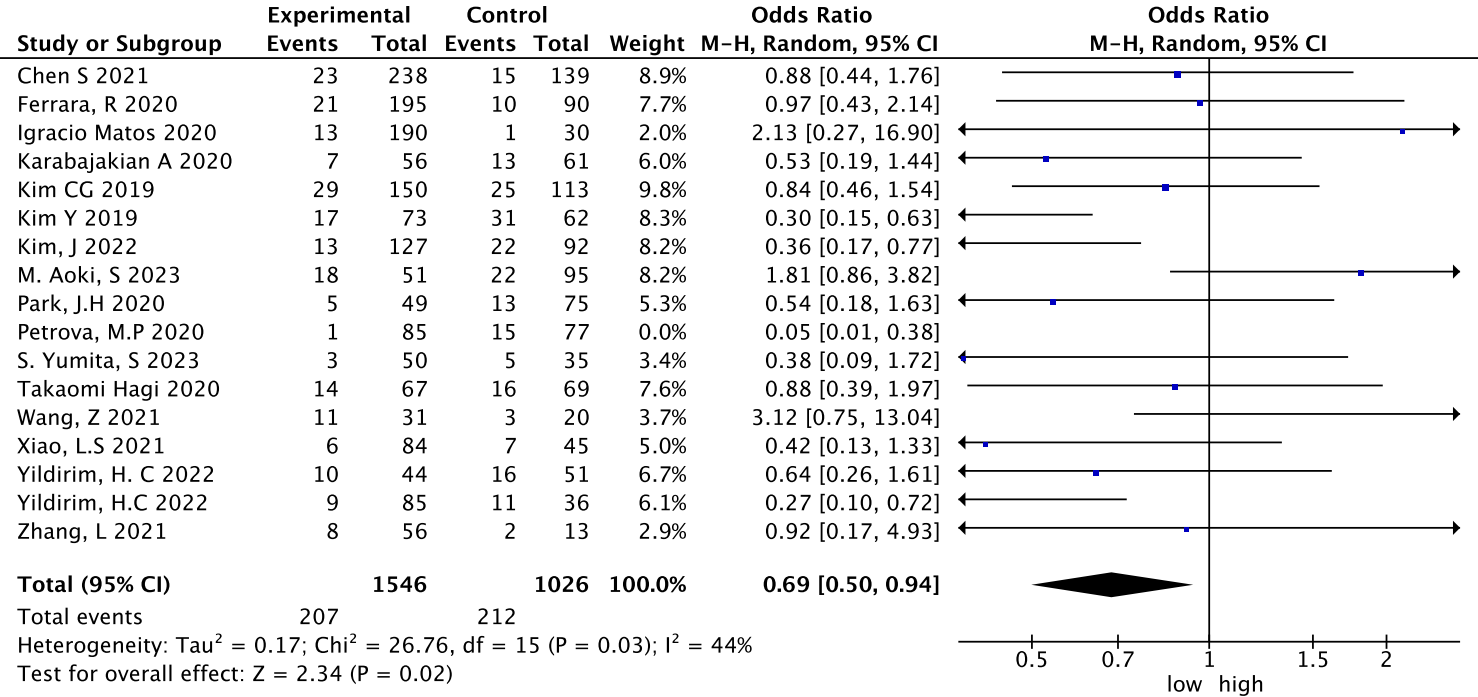

Supplement: Supplementary file 1 [file DataSheet1.zip › Supplementary Figure 1-17/Supplementary Figure 4.pdf]

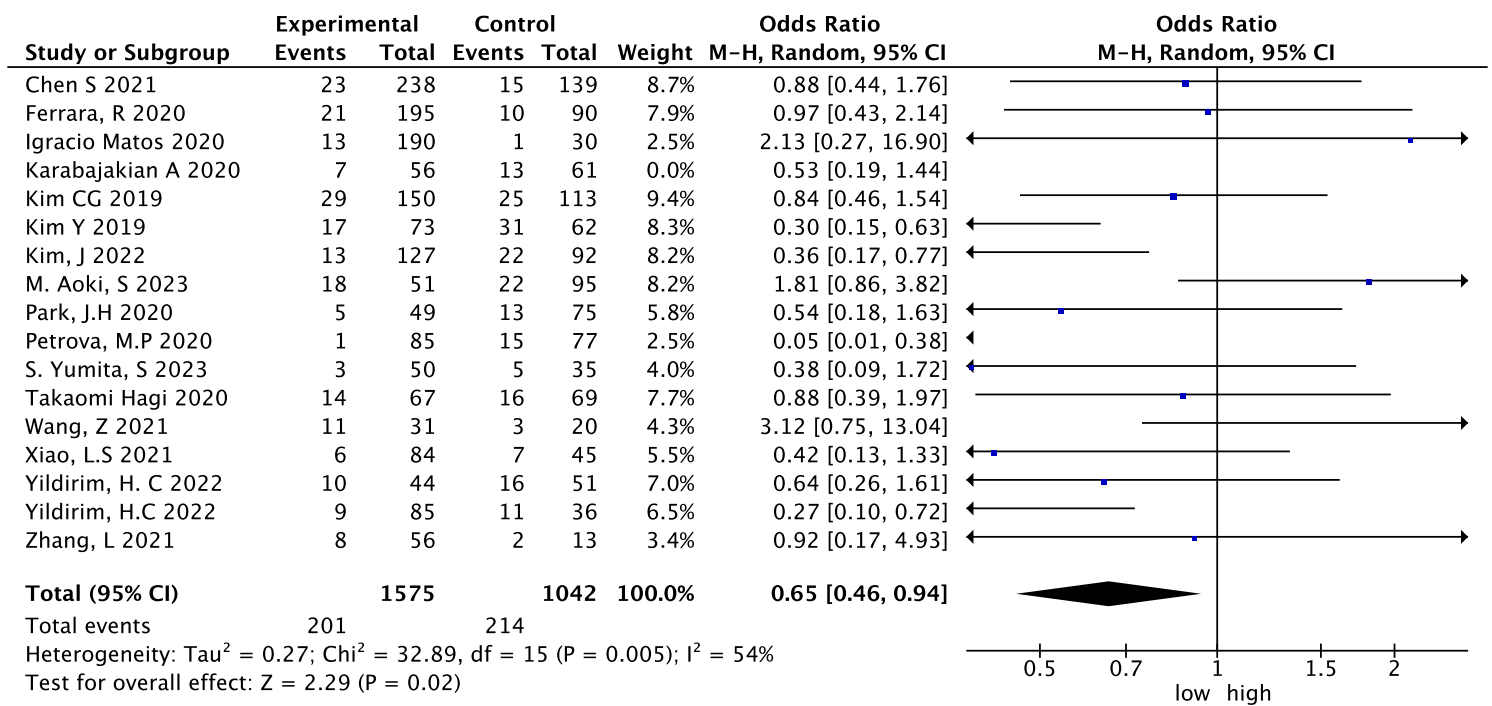

Supplement: Supplementary file 1 [file DataSheet1.zip › Supplementary Figure 1-17/Supplementary Figure 5.pdf]

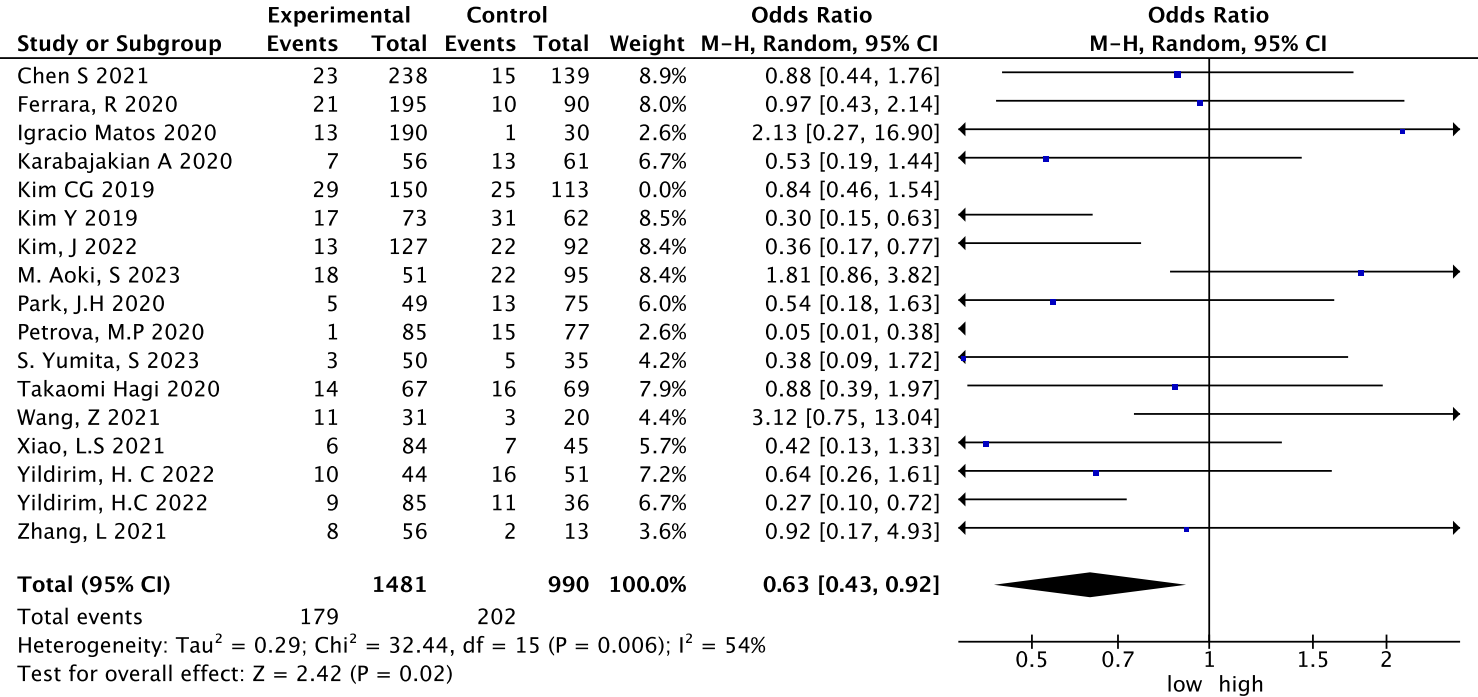

Supplement: Supplementary file 1 [file DataSheet1.zip › Supplementary Figure 1-17/Supplementary Figure 6.pdf]

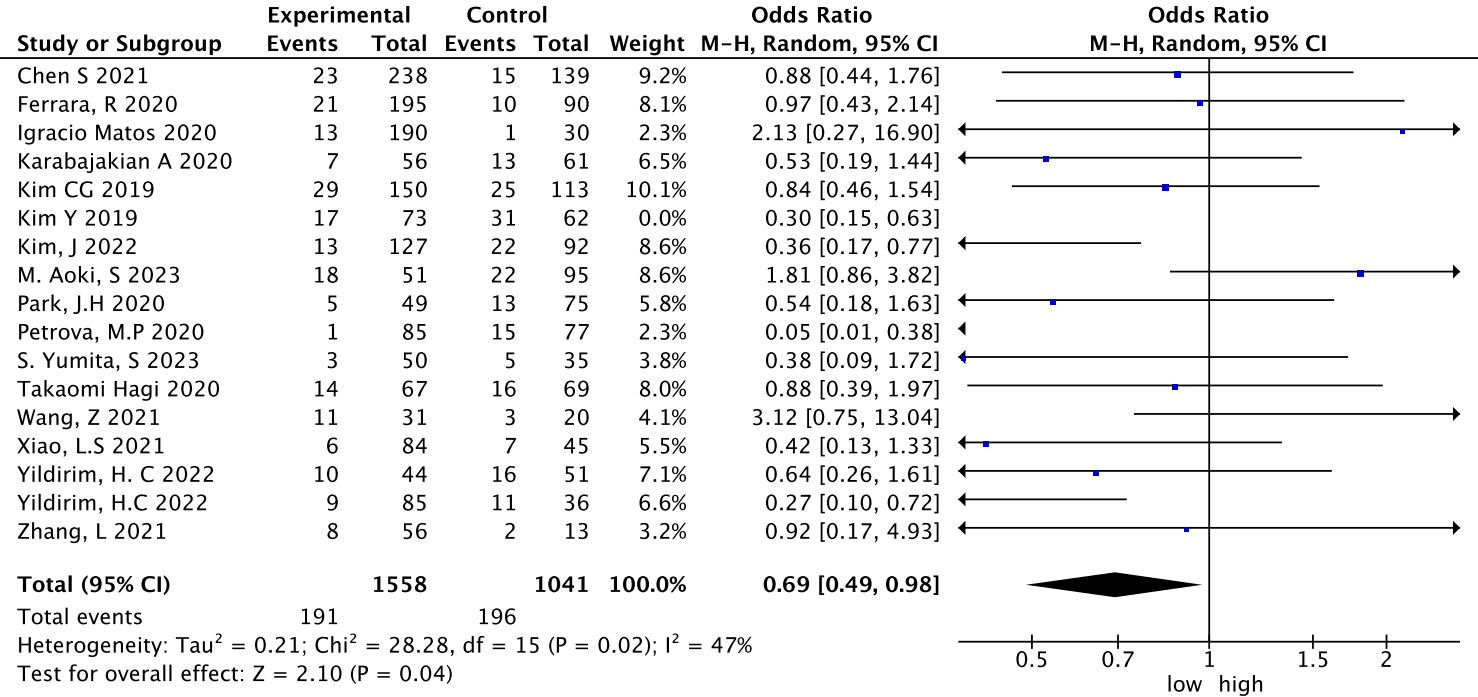

Supplement: Supplementary file 1 [file DataSheet1.zip › Supplementary Figure 1-17/Supplementary Figure 7.pdf]

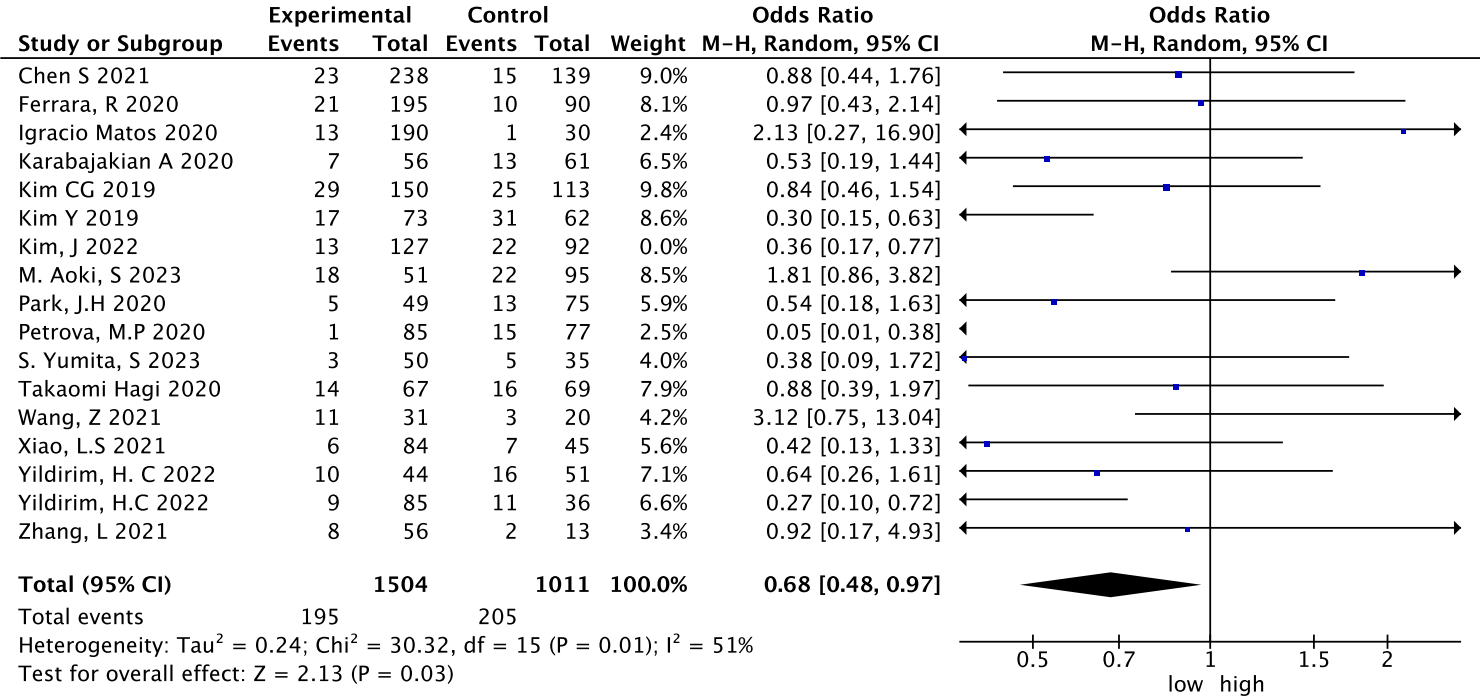

Supplement: Supplementary file 1 [file DataSheet1.zip › Supplementary Figure 1-17/Supplementary Figure 8.pdf]

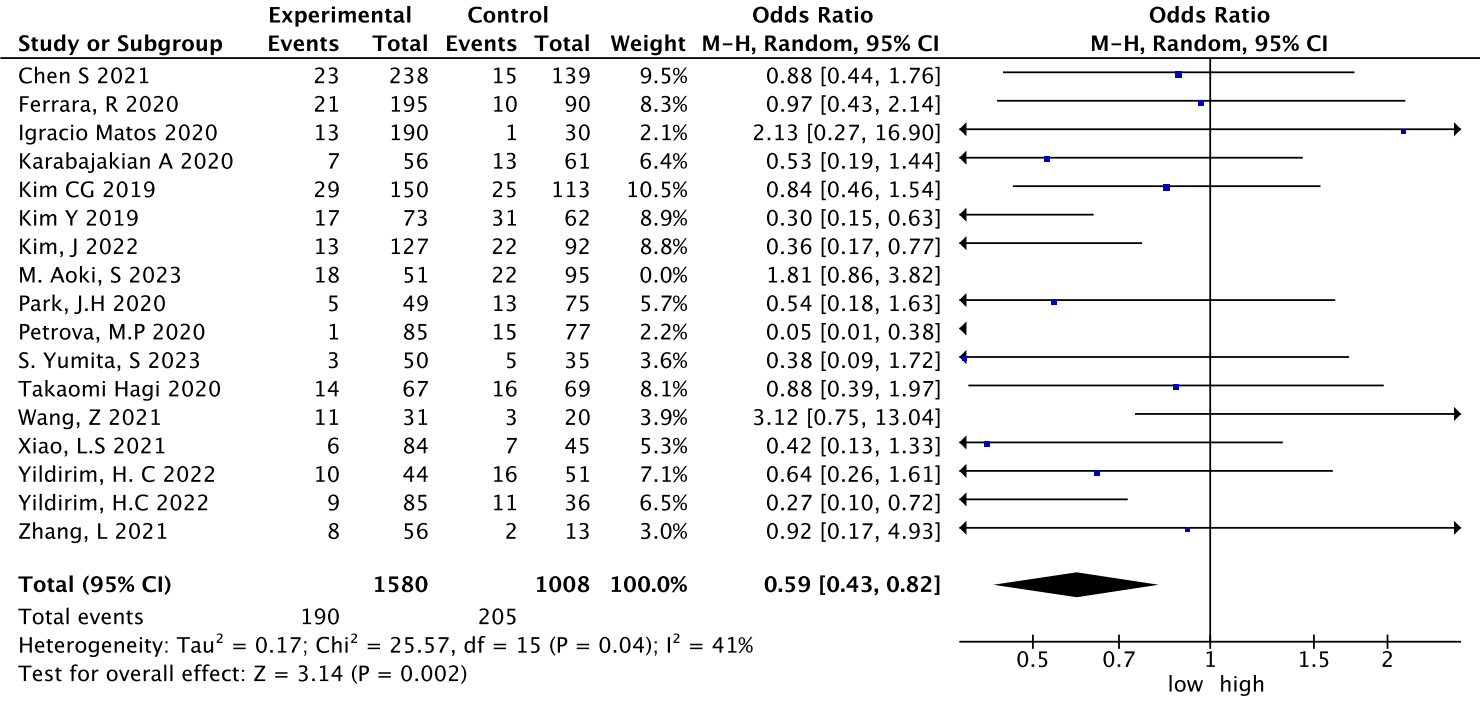

Supplement: Supplementary file 1 [file DataSheet1.zip › Supplementary Figure 1-17/Supplementary Figure 9.pdf]
